# Development of a Web-Based Clinical Decision Support System for Drug Prescription: Non-Interventional Naturalistic Description of the Antipsychotic Prescription Patterns in 4345 Outpatients and Future Applications

**DOI:** 10.1371/journal.pone.0163796

**Published:** 2016-10-20

**Authors:** Sofian Berrouiguet, Maria Luisa Barrigón, Sara A. Brandt, Santiago Ovejero-García, Raquel Álvarez-García, Juan Jose Carballo, Philippe Lenca, Philippe Courtet, MEmind Study Group, Enrique Baca-García

**Affiliations:** 1 Department of Psychiatry and Emergency, Brest Medical University Hospital, Brest, France; 2 Logics in Uses, Social Science and Information Science department, Telecom Bretagne, Plouzané, France; 3 Department of Psychiatry at Fundación Jimenez Diaz Hospital, Madrid, Spain; 4 Department of Psychiatry, Icahn School of Medicine at Mount Sinai, New York, NY, 10029, United States of America; 5 Department of Psychiatry, University Hospital Rey Juan Carlos, Móstoles, Madrid, Spain; 6 INSERM U888, Department of Psychiatry, Montpellier University Hospital, Montpellier, France; 7 Department of Psychiatry, IIS-Jimenez Diaz Foundation, Madrid, Spain; 8 Department of Psychiatry, University Hospital Infanta Elena, Valdemoro, Spain; 9 Department of Psychiatry, University Hospital Rey Juan Carlos, Móstoles, Spain; 10 Department of Psychiatry, General Hospital of Villalba, Autonoma University, Madrid, Spain; 11 CIBERSAM, Madrid, Spain; 12 Columbia University, New York, NY, United States of America; Maastricht University, NETHERLANDS

## Abstract

**Purpose:**

The emergence of electronic prescribing devices with clinical decision support systems (CDSS) is able to significantly improve management pharmacological treatments. We developed a web application available on smartphones in order to help clinicians monitor prescription and further propose CDSS.

**Method:**

A web application (www.MEmind.net) was developed to assess patients and collect data regarding gender, age, diagnosis and treatment. We analyzed antipsychotic prescriptions in 4345 patients attended in five Psychiatric Community Mental Health Centers from June 2014 to October 2014. The web-application reported average daily dose prescribed for antipsychotics, prescribed daily dose (PDD), and the PDD to defined daily dose (DDD) ratio.

**Results:**

The MEmind web-application reported that antipsychotics were used in 1116 patients out of the total sample, mostly in 486 (44%) patients with schizophrenia related disorders but also in other diagnoses. Second generation antipsychotics (quetiapine, aripiprazole and long-acting paliperidone) were preferably employed. Low doses were more frequently used than high doses. Long acting paliperidone and ziprasidone however, were the only two antipsychotics used at excessive dosing. Antipsychotic polypharmacy was used in 287 (26%) patients with classic depot drugs, clotiapine, amisulpride and clozapine.

**Conclusions:**

In this study we describe the first step of the development of a web application that is able to make polypharmacy, high dose usage and off label usage of antipsychotics visible to clinicians. Current development of the MEmind web application may help to improve prescription security via momentary feedback of prescription and clinical decision support system.

## Introduction

Since their first introduction in the 1950s, antipsychotic medications have been used to treat a growing array of conditions. While first approved as treatment for schizophrenia [1], their use has rapidly extended to other disorders. Currently, FDA-approved uses of typical antipsychotics include schizophrenia, bipolar disorder, psychotic disorders in general, agitation, hyperactivity, Tourette syndrome, generalized nonpsychotic anxiety, and severe behavioural problems [2]. The applicability of these drugs increased only further with the introduction of atypical antipsychotics [3]. These are now approved to treat conditions that range from autism to major depressive disorder (MDD) [4].

Nonetheless, off label use of antipsychotic drugs is still an extended practice. Prescribing antipsychotic drugs to treat unapproved conditions and employing excessive doses are two of the most common examples. In particular, antipsychotics have been regularly used to treat behavioural symptoms in elderly patients with dementia, despite conflicting evidence to support it [5]. In fact, the percentage of patients with FDA-unapproved disorders taking antipsychotics in the US has been estimated to range from 60% to 83% [6,7], with an estimated cost in 2008 of $6.0 billion [6].

Finally, many patients are treated with two or more antipsychotic drugs in combination, although its effectiveness remains to be demonstrated [8,9]. Antipsychotic polypharmacy refers to the co-prescription of more than one antipsychotic drug for an individual patient [10]. In outpatient settings, antipsychotic polypharmacy is somewhat less frequent when compared to inpatient populations, possibly due to lesser illness severity. However, in a large cohort in the US consisting of outpatients from three different health care settings, Sun et al. [11] reported that approximately one fifth of those with psychotic disorders were under a treatment schedule consisting of more than one antipsychotic drug. In Canada, Procyshyn et al. [12] reported an antipsychotic polypharmacy prevalence of 25.7% in outpatients. Furthermore, in a European outpatient setting, Novick et al. [9] reported polypharmacy in approximately one third of the patients, with a tendency for a slight increase during a one year follow up. A recent review further reported that among adults, off label prescription consisted of 40 to 75% of all antipsychotic prescriptions [13]. Antipsychotic polypharmacy is common. Evidence of efficacy however, is limited to small randomized controlled clinical trials, case reports, and individual clinician experience. At the same time, antipsychotic polypharmacy has been associated with an increased risk of metabolic syndrome [14], higher healthcare costs [15], and possibly mortality [16].

Over the last decade, medical prescription security has been supported by the emergence of electronic health records (EHRs) and clinical decision support systems (CDSS) that facilitate portability and processing of pertinent health information related to pharmacological treatment [17]. This has helped monitor prescription patterns that include off label use, polypharmacy and high dosage. Inter-institutional EHRs are used to further increase efficiency in medical services and provide complete and accurate medical information across providers in different institutions [18]. CDSS are made possible by digitalization of clinical data. Their purpose is to improve clinical management, methodological contributions, sensitivity and simulation tools in order to evaluate the clinical impact of the prescription decisions. The emergence of electronic prescribing devices with decision support systems significantly reduces error rates [19]. They also reveal an important source of epidemiological insight about how treatment is prescribed and taken, although these are extremely expensive and are not widely implemented. However, current EHRs often fall short of delivering readily available, compiled and tailored medical knowledge regarding the patient to the clinician [20]. The availability of handheld computing provides the opportunity to implement many of these gains to institutions where e-prescribing systems are not yet accessible. The increasing availability of smartphone technology permits the gathering of naturalistic data that can be processed immediately and provide instantaneous decision making assistance to clinicians.

A pioneer project in the 1980s programmed drug monitoring systems to identify evidence-based medication practices in 11 New York State Institutions for Mental Health and Developmental Disabilities. The results of the study showed that the surveillance techniques improved prescribing practices [21]. EHRs are now used routinely and are a major source of structured data. In a naturalistic, observational, retrospective, non-interventional study, Gavirina et al. [22] were able to describe prescription habits in a sample of 1700 patients suffering from schizophrenia. The data were gathered from computerized or e-medical records that were registered in the electronic medical record and implemented in all centers of the network that performed the assessment.

However, these systems present some limits that are related to the methodology of such studies; observational studies based on retrospective analysis of EHR data. For example routine practice data are collected for billing or institutional purposes. The re-use of these data to advance clinical research can be challenging [23]. The timing, quality, and comprehensiveness of clinical data are often not consistent with research standards [24]. Accuracy (correctness) of data relies on correct and careful documentation, which is often difficult to perform though most EHR used in routine. Furthermore, due to the architecture of traditional EHR, data cannot be processed instantaneously to deliver a CDSS, which requires a double task of de-identification of the data, and statistical analysis out of the software core. Most studies describing antipsychotic habits use retrospective methods [25]. By doing so, researchers and clinicians miss the opportunity to process gathered data in the moment and use them for CDSS purposes. The use of electronic records for decision support at a clinical level is still not widely reported. EHRs are usually commercial software only accessible from computers having wired connection. This characteristic excludes systematic assessment of outpatients that are treated by primary care services or those that are hospitalized out of the mainstream care services.

Taking into consideration the strengths and pitfalls of each of these monitoring strategies, we developed a web application that is able to adapt to any common routine follow-up strategy and research protocol in medicine. The growth and popularity of mHealth apps (health-related software applications), their ease of use and their cost of development compared to traditional software make them particularly suitable for the task of prescription monitoring.

Our Hypothesis was that a web application developed for this study may be able to describe clinician prescriptions. The objective of this study was to describe, via the Memind web-application, the prescription patterns of antipsychotics in a naturalistic outpatient setting in five Psychiatric Community Mental Health Centers in Madrid, Spain. We focused primarily on off label uses and antipsychotic polypharmacy.

## Materials and Methods

### Study setting

Four thousand nine hundred and seventy-five patients received psychiatric care in five Psychiatric Community Mental Health Centers (Moncloa Mental Health Center, Arganzuela Mental Health Center, Infanta Elena Hospital, Valdemoro Mostoles Hospital, Hospital 12 de Octubre Hospital) part of the Psychiatry Department of Fundacion Jimenez Diaz in Madrid, Spain, during the study period (from June 2014 to October 2014). This department is part of the National Health Service and provides medical coverage financed by taxes to a catchment area of 800,000 people. Fifty-five clinicians of the Memind study group participated in the patient recruitment process. Outpatients were assessed during routine medical visit by these clinicians. The clinician also created a profile in the MEmind web application for each patient that met the inclusion criteria and agreed to participate.

### Patient inclusion and exclusion criteria

Inclusion criteria were either male or female outpatients, aged 18 or older, who gave written informed consent. Participants were excluded from the study if they were under the age of 18, incarcerated, under guardianship, enrolled in other trials, or were in emergency situations where their state of health did not allow for obtaining written informed consent.

### Material

The web application was specifically developed for Android and OSX Smartphones, Tablets and the Mac and PC versions of Mozilla firefox and Google Chrome. It is available in three languages (English, French, Spanish). The web application has two distinct interfaces. The “electronic health record” view is designed for use by health care providers during clinical rounds and medical or nursing visits. It has been designed to cover all the data acquired during a standard psychiatric evaluation, including sociodemographic, diagnostic and pharmacological treatment information. Furthermore, it is based on commonly stored information in mental health management. A large customizable choice of relevant scales can be added by the care provider to the basic evaluation. The patient view was also developed and will allow patients to be monitored with ecological momentary assessment (EMA) tools in future studies. Patient had no access to this function for the present study.

**Fig 1:**
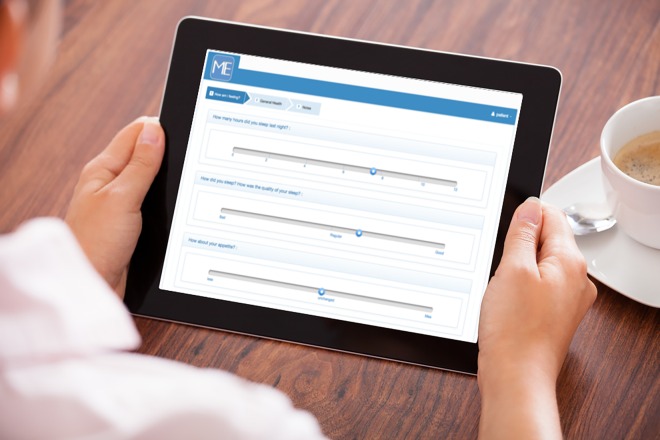
The MEmind web application as viewed from an OSX tablet.

Three different user profiles exist: 1) for patients; 2) for caregivers/family; and 3) for mental health professionals. For the purposes of this study we only used the mental health professional profile (Fig 1). During this study, patients did not have access to the web application. If they agreed to participate to the study, they only agreed to allow their clinician to enter their personal data into the MEmind web application. The patients were still treated as usual.

Each patient was identified by a numeric code that ensures patient anonymity. This code is encrypted in the database that remained the same throughout all contact with patients cared for during the study.

### Baseline assessment

For this study, variables collected for each patient profile were sex, age, diagnosis and treatment. Clinical diagnoses were made by psychiatrists or clinical psychologists, coded according to the ICD-10 [26] for mental disorders, and entered manually into the MEmind web-application. Diagnoses were assigned by the clinician, not automatically done by the application. Allocation of information was done by the clinician via the clinician interface of the web application.

### Outcomes measures

Pharmacological treatment was registered with our web tool and then classified according to the Anatomical Therapeutic Chemical (ATC) classification system and the defined daily dose (DDD)[27]. We calculated the average of daily dose prescribed for antipsychotics (N05A ATC code), Prescribed Daily Dose (PDD), and the PDD to DDD ratio. Although ATC classification includes lithium and antipsychotics under the N05A code, for the purpose of this study we only included antipsychotics. In order to compare dosages of the various antipsychotics we used a fixed unit of measurement based on dividing the prescribed daily dose (PDD) by the defined daily dose (DDD). A PDD/DDD ratio greater than 1.5 was defined as excessive dosing [12]. We performed double verification of the results using the SSPS version 22.0 package.

### Ethical considerations

The research was in compliance with the Code of Ethics of the World Medical Association (Declaration of Helsinki) and the standards established by the Institutional Review Board and granting agency. All the participants provided written informed consent, after the complete description of the study. Previously, the research protocol was approved by the local (Fundacion Jimenez Diaz) Ethics Committee.

## Results

### Diagnoses and sample characteristics

Out of the 4975 outpatients that received psychiatric care during study period, 4345 agreed to participate to the study. The distribution of psychiatric disorders attended in our outpatient units is shown in Table 1, and the distribution of the most common disorders according to age and gender is shown in Table 2.

**Table 1:** Prevalence of psychiatric disorders (n = 4975).

Psychiatric disorder	N (4975)	%
F0-F09 Organic, including symptomatic, mental disorders	85	2.0
F10-F19 Mental and behavioural disorders due to psychoactive substance use	314	7.2
F20-F29 Schizophrenia, schizotypal and delusional disorders	574	13.2
F30-F39 Mood [affective] disorders	1221	28.1
F40-F48 Neurotic, stress-related and somatoform disorders	1956	45.0
F50-F59 Behavioural syndromes associated with physiological disturbances and physical factors	176	4.1
F60-F69 Disorders of adult personality and behaviour	475	10.9
F70-F79 Mental retardation	41	0.9
F80-F89 Disorders of psychological development	10	0.2
F90-F98 Behavioural and emotional disorders with onset usually occurring in childhood and adolescence	74	1.7
F99-F99 Unspecified mental disorder	49	1.1

**Table 2:** Age and gender distribution of most common psychiatric disorders (n = 4354).

		Schizophrenia and other psychoses (n = 574; 13.2%)	Mood disorders (n = 1221; 28.1%)	Neurotic. stress related and somatoform disorders (n = 1956; 45.0%)	Personality disorder (n = 475; 10.9%)	Total (n = 4345)
Age n (%)	18–35 years	132 (23%)	146 (12%)	424 (21.7%)	114 (24.1%)	890 (20.5%)
	35–50 years	232 (40.5%)	329 (27%)	733 (37.5%)	208 (43.6%)	1517 (34.9%)
	50–65 years	160 (27.8%)	446 (36.5%)	561 (28.7%)	119 (25.1%)	1304 (30%)
	>65 years	50 (8.6%)	300 (24.5%)	238 (12.2%)	35 (7.3%)	634 (14.6%)
	p-value	<0.001	<0.001	<0.001	<0.001	
Gender n (%)	Female	377 (65.6%)	855 (70.0%)	1330 (68.0%)	275 (57.9%)	1621 (62%)
	Male	197 (34.4%)	366 (30.0%)	626 (32.0%)	200 (42.1%)	2724 (37.3%)
	p-value	<0.001	<0.001	<0.001	0.024	

Out of our total sample, 3640 patients had one psychiatric diagnosis, with anxiety and related disorders (F40-F49) being the more frequent diagnoses. The rest of the patients showed a co-morbid condition. Antipsychotics were used in 1116 patients (25% of total patients), 829 (74%) in monotherapy and 287 (26%) in poly-therapy (for detail see Table 3).

**Table 3:** Antipsychotic monotherapy and polypharmacy according diagnosis (n = 1116).

Diagnosis		Patient with specific diagnostic N (%)	Antipsychotic use	Antipsychotic monotherapy	Antipsychotic polypharmacy
One diagnosis	F0-F09	44 (1.0%)	26 (2.3%)	23 (2.8%)	3 (1%)
	F10-F19	111 (2.6%)	26 (2.3%)	24 (2.9%)	2 (0.7%)
	F20-F29	476 (11.0%)	462 (41.4%)	305 (36.8%)	157 (54.7%)
	F30-F39	966 (22.2%)	268 (24%)	204 (24.6%)	64 (22.2%)
	F40-F49	1630 (37.5%)	82 (7.3%)	78 (9.4%)	4 (1.4%)
	F50-F59	101 (2.3%)	9 (0.8%)	9 (1.1%)	0 (0%)
	F60-F69	203 (4.7%)	69 (6.1%)	60 (7.2%)	9 (3.1%)
	F70-F79	22 (0.5%)	13 (1.2%)	9 (1.1%)	4 (1.4%)
	F90-F99	87 (2%)	7 (0.62%)	7 (0.8%)	0 (0%)
Comorbidity	F0-F09 + F30-F39	15 (0.3%)	11 (1%)	8 (1%)	3 (1.0%)
	F10-F19 + F20-F29	23 (0.5%)	23 (2%)	11 (1.3%)	12 (4.2%)
	F10-F19 + F30-F39	33 (0.8%)	12 (1%)	8 (1%)	4 (1.4%)
	F10-F19 + F40-F49	36 (0.8%)	5 (0.4%)	5 (0.6%)	0 (0%)
	F10-F19 + F60-F69	23 (0.5%)	9 (0.8%)	7 (0.8%)	2 (0.7%)
	F20-F29 + F40-F49	13 (0.3%)	12 (1%)	10 (1.2%)	2 (0.7%)
	F20-F29 + F60-F69	13 (0.3%)	12 (1%)	6 (0.7%)	6 (2.1%)
	F30-F39 + F40-F49	47 (1.1%)	8 (0.7%)	6 (0.7%)	2 (0.7%)
	F30-F39 + F50-F59	17 (0.4%)	1 (0.1%)	0 (0%)	1 (0.3%)
	F30-F39 + F60-F69	83 (1.9%)	32 (2.9%)	22 (2.7%)	10 (3.5%)
	F40-F49 + F40-F49	73 (1.7%)	10 (0.9%)	10 (1.2%)	0 (0%)
	F40-F49 + F50-F59	32 (0.7%)	1 (0.1%)	1 (0.1%)	0 (0%)
	F40-F49 + F60-F69	73 (1.7%)	18 (1.6%)	16 (1.9%)	2 (0.7%)
	Other combinations	224 (5.2%)	0 (0%)	0 (0%)	0 (0%)
	TOTAL	4345	1116 (26%)	829 (19%)	287 (6.6%)

### Antipsychotic pattern use

Concerning diagnoses, as a simple diagnosis or comorbidity, out of the 1116 patients in which antipsychotics were prescribed, 44% of them (486 patients) were used in patients with schizophrenia and related disorders, 28% (309 patients) in affective disorders, 13% (140 patients) in personality disorders, and 136 (12%) in anxiety and related disorders. It is interesting to note that 14 patients with schizophrenia and related disorders did not receive antipsychotics (Table 3).

Out of the 1116 patients using antipsychotics, 1475 antipsychotic prescriptions were made. Out of the 829 patients (74%) using an antipsychotic as a monotherapy, antipsychotics were the only therapy used for 185 (185/829, 22%) of them. The most common combination of antipsychotics with another psychotropic drug was with antidepressants (in 109 patients). Antipsychotics were used in polypharmacy regimen in 287 patients (26%). In 151 patients, the combinations were only used with other antipsychotics.

Table 4 shows the different antipsychotics prescribed, ATC/DDD classification and doses employed in our clinical practice. It is important to note that antipsychotics used in an excessive dosing were long-acting paliperidone and ziprasidone.

**Table 4:** Prescribed Daily Dose (PDD), and the PDD to defined daily dose ratio of antipsychotics (n = 1116).

Number of prescriptions	Drug	ATC code	DD (mg)	Median PDD (mg)	Mean PDD (mg)	PDD (mg) IC 95%	PDD/DDD
47	Amisulpride	N05AL05	400	400.00	520.21	401.63–638.79	1.30
262	Aripiprazole	N05AX12	15	10.00	12.76	11.68–13.84	0.85
88	Asenapine	N05AH05	20	7.50	9.29	8.07–10.51	0.46
3	Chlorpromazine	N05AA01	300	100.00	76.67	30.93–122.40	0.26
24	Clotiapine	N05AH06	80	40.00	37.20	31.71–42.69	0.47
65	Clozapine	N05AH02	300	280.00	287.92	254.42–321.43	0.96
16	Fluphenazine	N05AB02	1	0.89	0.89	0.72–1.05	0.89
25	Haloperidol	N05AD01	8	5.00	6.96	4.36–9.55	0.87
13	Levomepromazine	N05AA01	300	38.75	63.61	41.46–85.76	0.21
1	Levosulpiride	N05AL07	400	25.00	25.00		0.06
150	Olanzapine	N05AH03	10	10.00	9.82	8.66–10.98	0.98
104	Paliperidone	N05AX13	6	6.00	7.56	6.61–8.50	1.26
214	Long-acting paliperidone	N05AX13	2,5	3.57	4.41	4.17–4.65	1.76
1	Perphenazine	N05AB03	30	8.00	8.00		0.27
3	Pimozide	N05AG02	4	4.00	3.67	0.82–6.51	0.92
279	Quetiapine	N05AH04	400	100.00	194.35	170.76–217.95	0.49
110	Risperidone	N05AX08	5	3.00	4.15	3.51–4.79	0.83
5	Long-acting risperidone	N05AX08	2,7	3.57	3.57	1.65–5.49	1.32
1	Sertindole	N05AE03	16	4.00	4.00		0.25
2	Sulpiride	N05AL01	800	100.00	100.00		0.13
19	Tiapride	N05AL03	400	100.00	144.74	107.34–182.13	0.36
14	Ziprasidone	N05AE04	80	90.00	130.00	72.36–187.64	1.63
1	Zuclopenthixol	N05AF05	30	25.00	25.00		0.83
28	Zuclopenthixol depot	N05AF05	15	9.52	11.39	10.01–12.78	0.76

The proportion of polypharmacy for every antipsychotic and the dose used when antipsychotics were used alone or in combination are showed in Tables 5 and 6.

**Table 5:** Proportion of antipsychotic polypharmacy compared with monotherapy (n = 1116).

Drug	ATC code	Prescriptions N (%)	Monotherapy N (%)	Polypharmacy N (%)
Amisulpride	N05AL05	47 (3.2%)	19 (40%)	28 (60%)
Aripiprazole	N05AX12	262 (17.6%)	181 (69%)	81 (31%)
Asenapine	N05AH05	88 (5.9%)	59 (67%)	29 (33%)
Chlorpromazine	N05AA01	3 (0.2%)	1 (33%)	2 (67%)
Clotiapine	N05AH06	24 (1.6%)	5 (21%)	19 (79%)
Clozapine	N05AH02	65 (4,4%)	22(34%)	43 (66%)
Fluphenazine	N05AB02	16 (1.1%)	5 (31%)	11 (69%)
Haloperidol	N05AD01	25 (1.7%)	11 (44%)	14 (56%)
Levomepromazine	N05AA01	13 (0.9%)	10 (77%)	3 (23%)
Levosulpiride	N05AL07	1 (0.1%)	0	1 (100%)
Olanzapine	N05AH03	150 (10.0%)	102 (68%)	48 (32%)
Paliperidone	N05AX13	104 (7%)	63 (61%)	41 (39%)
Long-acting paliperidone	N05AX13	214 (14.4)	118 (55%)	96 (45%)
Perphenazine	N05AB03	1 (0.1%)	1 (100%)	0
Pimozide	N05AG02	3 (0.2%)	2 (67%)	1 (33%)
Quetiapine	N05AH04	279 (18.8%)	176 (63%)	103 (37%)
Risperidone	N05AX08	110 (7.5%)	61 (55%)	49 (45%)
Long-acting risperidone	N05AX08	5 (0.3%)	4 (80%)	1 (20%)
Sertindole	N05AE03	1 (0.1%)	0	1 (100%)
Sulpiride	N05AL01	2 (0.1%)	2 (100%)	0
Tiapride	N05AL03	19 (1.3%)	15 (79%)	4 (21%)
Ziprasidone	N05AE04	14 (0.9%)	4 (29%)	10 (71%)
Zuclopenthixol	N05AF05	1 (0.1%)	0	1 (100%)
Zuclopenthixol depot	N05AF05	28 (1.9%)	5 (18%)	23 (82%)
TOTAL		1475	866 (58.7%)	609 (41%)

**Table 6:** Antipsychotic doses when use in combination (n = 287).

Drug	ATC code	One antipsychotic		Two antipsychotics		Three antipsychotics		Four antipsychotics	
		N (%)	Mean PDD (mg)	N (%)	Mean PDD (mg)	N (%)	Mean PDD (mg)	N (%)	Mean PDD (mg)
Amisulpride	N05AL05	19 (2.2%)	315.79	22 (4.5%)	665.91	5 (4.7%)	720.00	1 (10%)	200.00
Aripiprazole	N05AX12	181 (21%)	11.01	71 (14.4%)	16.48	9 (8.4%)	17.22	1 (10%)	25.00
Asenapine	N05AH05	59 (6.8%)	8.01	23 (4.7%)	12.61	6 (5.6%)	9.17	0 (0%)	
Chlorpromazine	N05AA01	1 (0.1%)	30.00	0 (0%)		2 (1.9%)	100.00	0 (0%)	
Clotiapine	N05AH06	5 (0.6%)	34.00	13 (2.6%)	35.38	5 (4.7%)	48.00	1 (10%)	20.00
Clozapine	N05AH02	22 (2.5%)	248.64	40 (8.13%)	307.13	3 (2.8%)	320.00	0 (0%)	
Fluphenazine	N05AB02	5 (0.6%)	0.63	8 (1.62%)	1.05	3 (2.8%)	0.89	0 (0%)	
Haloperidol	N05AD01	11 (1.3%)	3.75	11 (2.2%)	9.34	2 (1.9%)	12.50	1 (10%)	5.00
Levomepromazine	N05AA01	10 (1.2%)	76.00	2 (0.4%)	100.00	1 (0.9%)	100.00	0 (0%)	
Levosulpiride	N05AL07	0 (0%)		1 (0.2%)	25.00	0 (0%)		0 (0%)	
Olanzapine	N05AH03	102 (11.8)	8.97	37 (7.5%)	11.96	10 (9.3%)	11.00	1 (10%)	5,00
Paliperidone	N05AX13	63 (7.3%)	6.57	31 (6.3%)	8.81	9 (8.4%)	10.67	1 (10%)	3.00
Long-acting paliperidone	N05AX13	118 (13.6%)	4.00	81 (16.5%)	4.81	15 (14%)	5.48	0 (0%)	
Perphenazine	N05AB03	1 (0.1%)	8.00	0 (0%)		0 (0%)		0 (0%)	
Pimozide	N05AG02	2 (0.23%)	2.50	1 (0.2%)	6.00	0 (0%)		0 (0%)	
Quetiapine	N05AH04	176 (20.3%)	158.45	79 (16.1%)	244.78	21 (19.6%)	309.52	3 (30%)	166.66
Risperidone	N05AX08	61 (7.0%)	3.12	41 (8.3%°	5.30	7 (6.5%)	6.50	1 (10%)	3.00
Long-acting risperidone	N05AX08	4 (0.5%)	3.57	1 (0.2%)	3.57	0 (0%)		0 (0%)	
Sertindole	N05AE03	0 (0%)		1 (0.2%)	4.00	0 (0%)		0 (0%)	
Sulpiride	N05AL01	2 (0.2%)	100.00	0 (0%)		0 (0%)		0 (0%)	
Tiapride	N05AL03	15 (1.7%)	140.00	3 (0.6%)	150.00	1 (0.9%)	200.00	0 (0%)	
Ziprasidone	N05AE04	4 (0.5%)	65.00	7 (1.4%)	165.71	3 (2.8%)	133.33	0 (0%)	
Zuclopenthixol	N05AF05	0 (0%)		1 (0.2%)	25.00	0 (0%)		0 (0%)	
Zuclopenthixol depot	N05AF05	5 (0.6%)	9.52	18 (3.7%)	11.90	5 (4.7%)	11.43	0 (0%)	
TOTAL		866		492		107		10	

## Discussion

### Main findings

In this study, we described a method to perform naturalistic prospective data gathering regarding prescription habits via a web-based application available from a smartphone or any other Internet-connected wireless device. This observational study was the first step of the development of CDSS that may help care providers to better monitor their prescriptions. We found that antipsychotics were mostly used in schizophrenia and related disorders but also in other disorders, in both approved and off label indications. Antipsychotic polypharmacy was employed in 26% of prescriptions made. Second-generation antipsychotics were mainly chosen for prescriptions (quetiapine, aripiprazole and long-acting paliperidone). Excessive dosing was only found with long acting paliperidone and ziprasidone whereas the use of low doses was relatively common. In this study, we were able to identify polypharmacy high dose and off label use in an outpatient setting. These results provide information about drug management under real conditions and highlight discrepancies between guidelines and actual practice that could guide research in new uses for drugs.

### Limitations

The current study was performed in a naturalistic setting of patients treated as usual. Out of the 4975 outpatients that received psychiatric care during study period, 4345 agreed to participate to the study. It is likely that some patients were not asked to participate due to the supplementary burden of entering data into the MEmind web application. Furthermore, some patients did not meet the inclusion criteria or refused to participate. During the preliminary process of the study, clinicians pointed out that the monitoring of patient acceptability, including refusal and reason for refusal or agreement, would add an additional task to fulfill during the consultation. This could have altered the naturalistic setting required for the study. Another concern of clinicians was also that this monitoring would have provided information about individual performance toward recruitment process. For these reasons, patients not meeting the inclusion criteria, patients who were not asked to participate, and patients refusing to participate were considered to be “missing data”. Overall, 4345 were finally included in the final analysis. Thus, data were partially or totally missing for 630 patients (12%) that were not included in the final analysis.

### Type of antipsychotics used

In our practice, second-generation antipsychotics were more commonly used than classic antipsychotics, with quetiapine, aripiprazole and long-acting paliperidone representing the most prescribed antipsychotics. This prescription habit reflects how second-generation antipsychotics have become the first-line of treatment for schizophrenia because of their fewer extrapyramidal side effects [28–30].

The prevalent use of quetiapine reflects our outpatient setting with anxiety and mood disorders being the more frequent disorders. This also suggests greater approval and off label use of quetiapine in recent years [30]. In this way, quetiapine could be used for generalized anxiety disorder [31], depression [32,33], insomnia [34] or dementia-related psychiatric symptoms [30,35].

### Range of diagnoses where antipsychotics were used and off label use

Consistent with findings in Spain [36], in our sample, around half of antipsychotic prescriptions were made in schizophrenia and related disorders while the rest were in other diagnoses. We also found that antipsychotics were prevalently utilized for mood disorders, personality disorders and, anxiety and related disorders (F40-F49 IDC codes) with more than 50% of prescriptions used in these three categories. As mood disorders include bipolar disorder, antipsychotic use is logical and expected. It is also important to remember approved and evidence based use of second generation antipsychotics in treatment-resistant depression (29,30). For anxiety and personality disorders, mainly represented by borderline personality disorder, antipsychotic use is not approved but widespread, with evidence based results on the topic [37,38]. A minority use of antipsychotics in our sample was made by participants with substance use disorders, mental retardation or ICD codes between F0 and F09 which includes dementia. These are uses consistent with those previously reported [39,40]. Patients with schizophrenia that did not have antipsychotic medication were patients in a change in treatment process (wash out period).

Considering the prevalent use of antipsychotics, it is interesting to reflect on how within complex situations, or even just daily clinical practice, solutions may be required that sometimes do not fit guidelines. Additionally, it is common in psychiatric practice to treat symptoms against diseases using the pharmacodynamic properties of drugs in order to guide treatment [41].

### Antypsychotic polipharmacy

The 26% rate of antipsychotic polypharmacy found in our sample is consistent with rates in outpatient settings worldwide [9,11,12] and in Spain [42,43]. Guidelines accepted antipsychotic polypharmacy in cases of cross-titration, control of acute disturbances and clozapine augmentation [44,45]. As our study is a transversal observation of our sample, we cannot measure cases of cross-titration. Acute disturbances are more likely treated in an inpatient setting, so that is not a plausible explanation for our rates of polypharmacy. Concerning clozapine augmentation, we used this antipsychotic in polypharmacy in 66% of cases, but this still does not explain all the polypharmacy employed.

Interestingly, classic long acting antipsychotics were mostly used in polypharmacy, 82% of prescriptions of zuclopenthixol depot and 69% of fluphenazine, which may reflect a frequent and non-evidence based clinical practice in patients with chronic schizophrenia course [46].

Clotiapine is another classic antipsychotic more frequently used in combination (79% of prescriptions). This high rate of polypharmacy and the low dosage generally used of this drug (PDD/DDD = 0.47) probably highlights its use for its hypnotic properties rather than as an antipsychotic. Clotiapine is an antipsychotic with a rapid onset of action and a strong sedative effect, which explains its use in insomnia in psychotic and non-psychotic patients [47].

Finally, amisulpride is also used in combination (68%) more frequently than in monotherapy. It is commonly said that for amisulpride, polypharmacy is the rule rather than the exception [48]. Moreover, amisulpride may be particularly suitable for clozapine augmentation [49]. This is a probable use in our sample according to our data.

### Antipsychotics dosing

According to our results, only two antipsychotics were employed at excessive dosing: long-acting paliperidone (PDD/DDD = 1.76) and ziprasidone (PDD/DDD = 1.63). This finding is consistent with previous knowledge; high doses are prevalent in hospitalized patients with schizophrenia [50,51] and less frequent in outpatient settings [52].

On the other hand, many antipsychotics were also used at low doses. This dosage use could be explained by the outpatient setting, where patients usually are in an established moment of the illness. Furthermore, antipsychotics are used in conditions other than psychosis, like insomnia in the case of clotiapine, chlorpromazine or levomepromazine, or mood and anxiety disorders or behaviour disorders in dementia, in the case of quetiapine. Special mention is needed for asenapine, an antipsychotic approved in Spain only for manic episodes in bipolar disorder. This narrow indication probably reflects its low dose usage.

Information for this study was obtained with a novel web tool, the mental state tracker MEmind. This device also has an interesting potential use in clinical research. Data collected by the provider are instantaneously transmitted to the database and made available for data mining. The system is also able to deliver alarms. In this pilot study we only made both raw data and graphic information about medical prescription available for the provider. We are currently assessing the possibility of incorporating, in the analysis, data proceeding directly from the patient that uses the EMA function of the program.

### Future development and implementation

In this study, treatment related information was collected via the web-application but processed secondarily by statistical software. As a result, it would have been possible to give instantaneous feedback to the care provider. Programs also exist that are able to alarm care providers about treatment interaction [53]. However, for the first step of the development of our web application, we decided to only monitor prescription habits and disable momentary feedback about treatment and alarm function of our web application. Alarms and feedback are part of the decision making support process [54]. By enabling this function in our web application, we probably would have faced the professional reluctance to welcome our web application in a routine clinical setting. Many studies have encountered difficulties incorporating EHRs into routine practices, especially when associated with CDSS. We are currently assessing professional acceptability of the device in order to propose a CDSS to clinicians that could be easily incorporated in their practice. Evaluating changes in routine practice caused by the use of the Memind application would also be an objective.

The objective of this study was to describe the prescription habits of antipsychotics via the Memind web-application. The Memind web application has Patient interface but this feature was not enabled for the present study. As a result patient acceptability of the application was not assessed, and neither was treatment adherence or symptom tracking using the EMA function. EMA involves repeated sampling of subjects’ behaviors and experiences in real time, in their natural environment. EMA has been successfully used for real-time self-reporting of symptoms and behaviours. This feature will soon allow us to integrate data proceeding from patient self-monitoring into CDSS.

Although many EHR CDSS have yet to be developed, their performance varies largely. Approaches that foster the impact of CDSS often rely on improved alerting concerning evidence-based recommendation or drug-drug interaction. We also believe that alerting could be taken into account by implementing or refining a severity grading. For this very first step of development, we only focused on the capacity of the web application to monitor prescription [55]. However, smartphone technology gives us the opportunity to combine pharmacological insight into analysis with idiosyncratic clinical data proceeding either from medical assessment or patient. A promising challenge would be to further incorporate an alert algorithm into a clinical dimension, for example integrating it into EMA data [56].
